# Comparison of general and aesthetic effects between flapless and flap techniques in dental implantation: a meta-analysis of randomized controlled trials

**DOI:** 10.1186/s40729-021-00380-5

**Published:** 2021-10-01

**Authors:** Xiaomeng Gao, Siyu Qin, He Cai, Qianbing Wan

**Affiliations:** State Key Laboratory of Oral Diseases, National Clinical Research Center for Oral Diseases, Department of Prosthodontics, West China Hospital of Stomatology, Sichuan University, No. 14, Section 3, South Renmin Road, Chengdu, 610041 People’s Republic of China

**Keywords:** Flapless, Flap, Aesthetics, Dental implants, Meta-analysis

## Abstract

**Background:**

Information about the aesthetic effects of flapless in implant surgeries is scant. Differences of the survival rate (SR) and crestal bone loss (CBL) between the two techniques were also controversial. Thus, this review was aimed to compare the general and aesthetic effects of flapless and flap approaches in implant surgeries.

**Materials and methods:**

Following the principals of PRISMA, literature databases were searched for the eligible randomized controlled trials (RCTs) comparing the clinical performances of flap and flapless techniques. After that, relevant data of selected studies were pooled and analyzed to compare SR, bleeding on probing (BOP), probing depth (PD), visual analogue scale (VAS), papillae presentation index (PPI), keratinized mucosa (KM) width and CBL between the two techniques.

**Results:**

Fourteen RCTs were included. No significant difference was found in SR (RR = − 0.01, 95% confidence interval (CI) (− 0.05, 0.04)), BOP (OR = 0.40, 95% CI (0.15, 1.02)), KM width (WMD = − 0.42, 95% CI (− 1.02, 0.17)) between two groups. Subgroup analysis revealed that the difference of CBL was insignificant in two groups (WMD = − 0.13, 95% CI (− 0.63, 0.38)). However, flap techniques would lead more peri-implant PD (WMD = − 0.37, 95% CI (− 0.51, − 0.23)). Subgroup analysis also indicated lower VAS scores in flapless group after 1 day (WMD = − 1.66, 95% CI (− 2.16, − 1.16)) but comparable pain experience after 3 days (WMD = − 0.59, 95% CI (− 1.33, 0.16)). Mean difference of PPI (WMD = 0.32, 95% CI (0.28, 0.35)) between the two groups was significant.

**Conclusions:**

The flapless procedure showed a superiority in preserving gingival papillae, reducing postoperative pain and peri-implant PD compared to the flap procedure, while exhibiting comparable effects on SR, BOP, KW width changes and CBL. Flapless technique is more recommended at the ideal soft and hard tissue implanting sites.

## Introduction

Implant-supported restorations have become the primary treatments for missing teeth with great prognosis [1–3]. However, the long-term clinical performances of dental implants could be affected by many factors, such as clinicians’ experience, hard and soft tissue conditions of patients and surgery procedures.

Gaining access to the alveolar bone is an indispensable step of the implant surgical procedures. The traditional way to expose the bone was the flap technique with mucosa incision and flap elevation, which makes the surgery field more visible and allows guided bone regeneration. There are some flap surgical options depending on incision sites, whereas most of them would have the risks of leaving scars on the gingiva, and even disrupting vasculature. Besides, horizontal incision may also impair the normal gingival papillae form. [4]

Flapless technique is a modified way to conduct implant procedures and it did not involve horizontal or vertical incisors for immediate and delayed implant placement. [5] Usually, the flap elevation step was omitted or the entrance to bone was created by a tissue punch device, drill preparation or immediate implant placement (IIP) [6]. Flapless procedure is considered as a more non-invasive approach to alveolar bone as there is no incision to cut the blood supply from bone membranes or soft tissues. Insufficiency of blood supply may result in poor bone regeneration or integration around implants [7]. There are some drawbacks of this technique though. The lack of visibility may result in a compromised implant placement. Since the punch devices are commonly narrower than implants, possible overheating during preparation is worth noting [8].

Some experts have compared the clinical performances of the two techniques mainly focusing on the SR or success rate of implants, peri-implant marginal bone loss or KM width. Those conclusions of the existing investigations were conflicting, while most of them found comparable clinic effects with similar SR between the two techniques [9–11]. However, a study discovered that flapless technique would increase the failure risks of implants [12]. Another analysis also showed that flapless procedure was more effective in preserving bone width and height [13].

To date, the comparison of aesthetic effects between the two techniques has not been investigated systematically. Implantation in the aesthetic zoom is an elaborate and complicated procedure. Horizontal incisors between adjacent teeth may damage gingival papillae and decrease the height of papillae after crown restoration. On the other side, flapless technique would remove more keratinized mucosa which is also of great importance for implant success and aesthetics [14]^15^. Jungwon Lee and his colleagues have revealed that flapless ridge preservation exhibited effectiveness in preserving bone width, bone height, and KM width [13]. No consensus has been reached on the benefits of flapless or flap techniques on aesthetic outcomes, soft and hard tissue alterations after surgery so far.

Here, we conducted a meta-analysis and systematic review of the effects of flapless and flap techniques on general and aesthetic clinical performances. After searching electronic databases and screening of the eligibility of searched studies, 14 RCTs were included for further analyses. The SR, BOP, PD, VAS, PPI, KM width and CBL were analyzed systematically to assess the general and aesthetic effects.

## Materials and methods

The current study was conducted following the principles of Preferred Reporting Items for Systematic Reviews and Meta-analyses (PRISMA) guidelines [16]. A detailed protocol was developed and registered in advance in the PROSPERO (http://www.crd.york.ac.uk/PROSPERO/) (registration number: CRD4202019721).

Participants–Interventions–Comparisons–Outcomes–Study Design (PICOS) Question.

Participants: systematically healthy patients with loss of teeth in need of implant placement or participants in need of immediate implant placement (IIP).

Interventions: application of flapless techniques.

Comparisons: application of flap techniques.

Outcomes: general clinical outcomes like SR, BOP, peri-implant PD, and VAS; and aesthetic outcomes like PPI, KM width, and CBL.

Study design: RCTs only.

Thus, the study was designed to address the question ‘‘among patients treated with a flapless approach compared with flap implant placement, how did general and aesthetic clinical performances differ?’’.

### Eligibility criteria

According to the PICOS question, the studies satisfied the following inclusion and exclusion criteria were included in the present review.

Inclusion criteria:

(i) Studies with patients in need of implants placement. Patients have no systematic diseases, or the women in post menopause period.
(ii) The studies should include flap and flapless techniques and compare at least one general or aesthetic outcome.
(iii) Each study should contained at least 10 patients with minimal follow-up of 3 months.
(iv) Randomized controlled clinical trials are included only.

Only RCTs were included in our research as to guarantee the high-quality outcomes to draw a convincing conclusion. Besides, any studies including the animals or the cadaver specimens were excluded. The duplicated studies, letters, case reports, case series and reviews have been excluded.

### Search strategies and study selection

PubMed, Web of Science, Cochrane Library were searched for the eligible researches by two independent authors (X G and S Q) until 1st August, 2020. Key words used were as follows: “(((flapless and open flap surgery) OR (flap and full thickness and dental implant)) OR (flap and flapless and dental implant)) OR (flapless and flap and dental implant)”. For “grey” literature, the ClinicalTrials.gov (ClinicalTrials.gov) and the International Clinical Trials Registry Platform (https://www.who.int/clinical-trials-registry-platform) were searched for unpublished clinical studies or registries.

Firstly, the titles and abstracts of the eligible studies were imported to EndNote X8 (Thomson ResearchSoft, Standford, US) and screened according to the inclusion and exclusion criteria above. For the incomplete information in the titles or the abstract and the possible literatures qualified for the criteria, the full texts of articles were obtained. A third author (H C) was included in the process when disagreement arose and a consensus was made after discussion.

### Data extraction

A standard data extraction sheet was created by digging the information of each qualified article. Detailed data of each article pertaining to author names, year, sample size, follow-up time, outcomes, loss of follow-up, etc., were listed.

### Quality assessments of included studies

The risk of bias within each included RCT were graded by two independent authors (X G and S Q) based on the Cochrane Collaboration’s tool for assessing risk of bias for RCTs. Quality assessments was evaluated as “low”, “high”, or “unclear” through seven aspects including random sequence generation, allocation concealment, blinding of participants and personnel, blinding of outcome assessment, incomplete outcome data, selective reporting, and other bias.

### General clinical measurements

Primary outcomes included the results of SR, BOP, peri-implant PD, VAS.

The survival of implants was defined as the implant remaining in situ without mobility or fractures [17]. BOP was defined as presence of bleeding on gentle probing (0.15Ncm) and recorded on a binary scale (presence/absence) for each implant surface [18, 19]. Peri-implant PD was the distance measured from the mucosal margin to the bottom of the probable pocket to assess the peri-implant diseases [20, 21]. Pain and discomfort was also measured using a 10 cm VAS ranging from 0 (no pain) to 10 (worst pain imaginable) [22]. VAS data were analyzed into two subgroups (1 and 3 days), since the patients’ subject feeling is of great importance in implant treatments.

### Aesthetic outcomes

Secondly, we assessed the aesthetic outcomes including PPI, KM width and CBL. PPI was evaluated in the papillae between the implant and adjacent teeth (0 = no papilla, 1 = less than half, 2 = more than half but not complete, 3 = complete fill, and 4 = overfill). Three studies have compared the PPI index and one study has assessed both the mesial and distal PPI. In this case, only the mesial ones were included in the analysis as the mesial part of gingival papilla plays a vital role in the aesthetic outcomes [23]. KM width was measured from the mucogingival junction to the free gingival margin. The KM width in 3 months follow-up was included in the analysis [24]. Vertically CBL data from medial axial section of the implant were extracted, as three flapless techniques were included in our analysis. A correlation between different flapless procedures (IIP vs. punch/drilling) and the marginal bone loss was found after flapless surgeries in comparison the clinical effects of flapless with flap techniques [10]. Two subgroups, immediate and delayed implantation, were detected in the comparison of CBL between the two groups. The immediate group contained CBL results extracted after 6 months and the delayed included results at least 3 months after surgery.

### Synthesis of results

The data obtained above were all analyzed through Review Manager version 5.3 (The Cochrane Collaboration, Copenhagen, Denmark).

SR and BOP were imported as dichotomous data, where the numbers of events in each group were extracted to evaluate the risk ratio (RR) and odds ratio (OR), respectively, in their 95% CI. The rest of the data were presented as means and standard deviations and analyzed as continuous figures to compare their weighted mean differences (WMD) and 95% CI. The mean differences were considered significant as *P* < 0.05. Considering the sample differences and heterogeneity of included studies, either a fixed or a random effects model was indicated and utilized.

### Assessment of publication bias

The publication bias across studies was evaluated using the Egger’s test by Stata SE release15 (StataCorp LP, College Station, TX, US) [25].

### Additional analysis

Sensitivity analyses were performed to determine if the heterogeneity of the outcomes were dependent on any individual study. The Stata SE release15 was utilized to investigate the impact of removing each of the selected studies.

To compare VAS scores within 3 days, qualified data were divided into “1 day” and “3 days” subgroups. Moreover, to evaluate the correlation of CBL using flapless or flap techniques after immediate or delayed implant surgery, studies included were stratified into “immediate” and “delayed” subgroups.

## Results

### Study selection

**Fig. 1:**
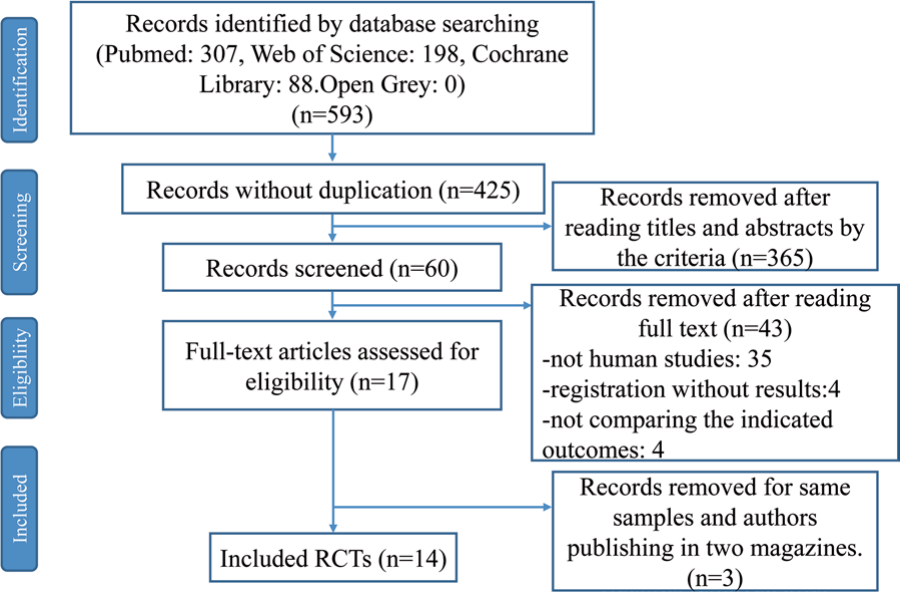
Flowchart of included studies

Initially, 593 studies were screened from PubMed, Web of Science, Cochrane Library and Open Grey with 168 duplicated articles and 3 articles investigating the same participants on different time. After the screening, only 14 studies satisfied the inclusion criteria and were included in the following investigation (Fig. 1) [23, 26–38].

### Study characteristics

Detailed information of selected studies is listed in Table 1. A total of 720 implants were included in the analysis. Among the 14 included studies, two studies comprised smoking patients; nine studies included only delayed implant and four studies included only immediate implants, whereas one study included both delayed and immediate implantation with no further analyses about the effects of the implant timing on the outcomes. Twelve studies have more than one year follow-up time and the rest of them also showed at least 6-month follow-up time. Only three studies included implants in the aesthetic zone (anterior teeth and premolars). Most of the studies compared the results in two group people, with two studies designing the one-split RCTs. One study evaluated the outcomes of the flapless technique with one-piece implants and flap technique with two-piece implants. All of the three studies comparing PPI contained only delayed implant placement. Most of the studies have measured the marginal bone changes, but only the vertical bone changes were included.

**Table 1:** Characteristics of the included studies

Studies	Size (implants)	Location	Implantation time	Flapless technique	Implant type	Follow-up time	Outcomes (flapless vs flap)							Loss of follow-up
							PPI	CBL (mm)	KW width (mm)	SR (100%)	BOP (100%)	Peri-implant PD	VAS	
Wadwa 2015 [25]	32	Mandibular first molar	Delayed	Punch	NR	0,3,9,15 m	NR	0.02 ± 0.01 VS 1.42 ± 0.09	NR	NR	NR	NR	NR	0
Bashutski 2013 [26]	24	Maxillary anterior or premolar tooth	Delayed	Punch	Two-piece	0,3,6,9,15 m	2.57 ± 0.46 VS 2.28 ± 0.46	NR	3.33 ± 0 .74 VS 3.97 ± 1.59	NR	NR	NR	NR	0
Kumar 2018 [27]	20	Posterior mandible	Delayed	Punch	NR	0.6.12 m	NR	NR	NR	NR	NR	1.40 ± 0.50 VS 2.00 ± 0.70	1.33 ± 0.50 VS 3.11 ± 1.17 (1 day) 0.33 ± 0.50 VS 0.67 ± 0.50 (3 days)	2
Wang 2017[28]	40	Mandibular first molar	Delayed	Drill preparation	One-piece	0,1,3,6,12,24 m	NR	0.70 ± 0.30 VS 0.50 ± 0.40	3.70 ± 1.10 VS 4.00 ± 1.30	100 VS 100	NR	2.20 ± 0.05 VS 2.70 ± 0.02	NR	0
Sunitha 2013 [29]	40	Anterior and premolar region	Delayed	Drill preparation	Two-piece	0,6,12.24 m	2.75 ± 0.05 VS 1.95 ± 0.06	0.03 ± 0.01 VS 0.20 ± 0.06	NR	NR	NR	NR	NR	0
Grassi 2019 [30]	30	Maxillary premolar area	Immediate	IIP	Two-piece	0,6 m	NR	NR	NR	NR	NR	NR	5.32 ± 1.21 VS 7.51 ± 0.98 (1 day) 1.82 ± 1.13 VS 2.98 ± 1.82 (3 days)	1
Tsoukaki 2013 [31]	30	Full dentition	Delayed	Punch	One-piece	1, 2, 6, and 12 weeks	NR	0 vs 0.29 ± 0.06	NR	NR	NR	1.93 ± 0.06 VS 2.43 ± 0.06	0.68 ± 0.22 VS 2.06 ± 0.22 (1 day)	0
Stoupel 2016 [32]	39	Maxillary anterior region	Immediate	IIP	Two-piece	3,6,12 m	NR	0.89 ± 0.80 VS 0.95 ± 0.92	NR	NR	NR	NR	NR	3
Froum 2017 [22]	28	Full dentition	Delayed	Punch	One-piece	8.2y	2.70 ± 0.80 VS 2.67 ± 0.75	0.25 ± 0.25 VS 0.70 ± 0.25	NR	100 VS 100	15.38 VS 21.43	2.10 ± 0.40 VS 2.40 ± 0.30	NR	1
Yang 2017 [33]	160	Maxillary anterior region	Immediate	IIP	NR	12 m	NR	NR	NR	NR	6.25 VS 16.25	1.72 ± 0.30 VS 1.76 ± 0.33	NR	0
Covani 2008 [34]	20	Full dentition	Immediate	IIP	Two-piece	0.6 m	NR	0.80 ± 0.90 VS 0.30 ± 0.40	NR	NR	NR	NR	NR	1
Bömicke 2017[35]	38	Posterior mandible	Delayed	Punch	Flapless: one-piece; flap: two-piece	0,12,36 m	NR	0.86 ± 0.63 VS 0,63 ± 0.43	NR	94.74 VS 100	NR	2.90 ± 0.65 VS 3.40 ± 0.70	NR	3
Cannizzaro [36]	143	Any two separate edentulous areas	Delayed and immediate	Drill preparation or IIP	NR	0,12 m	NR	0.24 ± 0.29 VS 0.33 ± 0.50	NR	97.22 VS 97.18	NR	NR	NR	4
Pisoni 2016[37]	76	Premolar and molar sites	Delayed	Punch	NR	0, 2 (lower) or 3 (upper), 36 m	NR	0.20 ± 0.76 VS 0.17 ± 0.94	NR	NR	NR	NR	NR	7

*NR* not reported, *IIP* immediate implant placement, *m* months

### Risk of bias within included studies

**Fig. 2:**
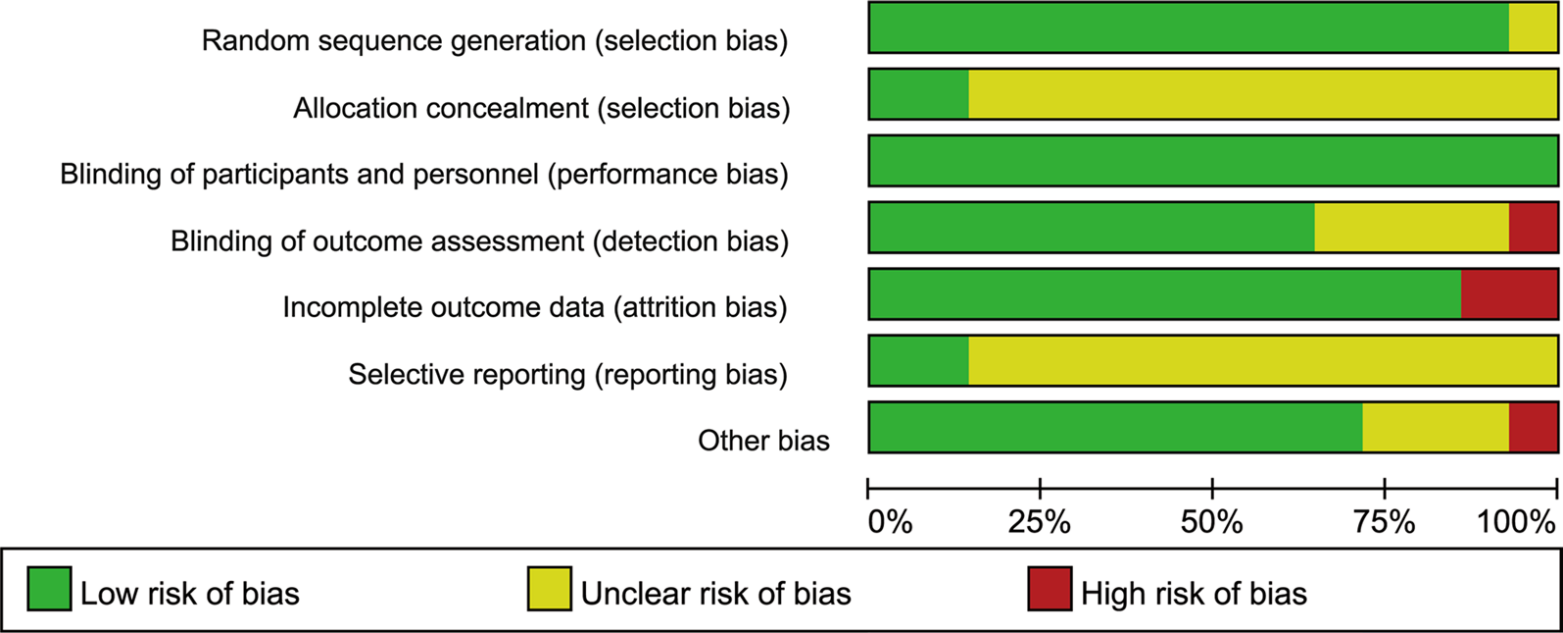
Bias items presented as percentages across all included studies

**Fig. 3:**
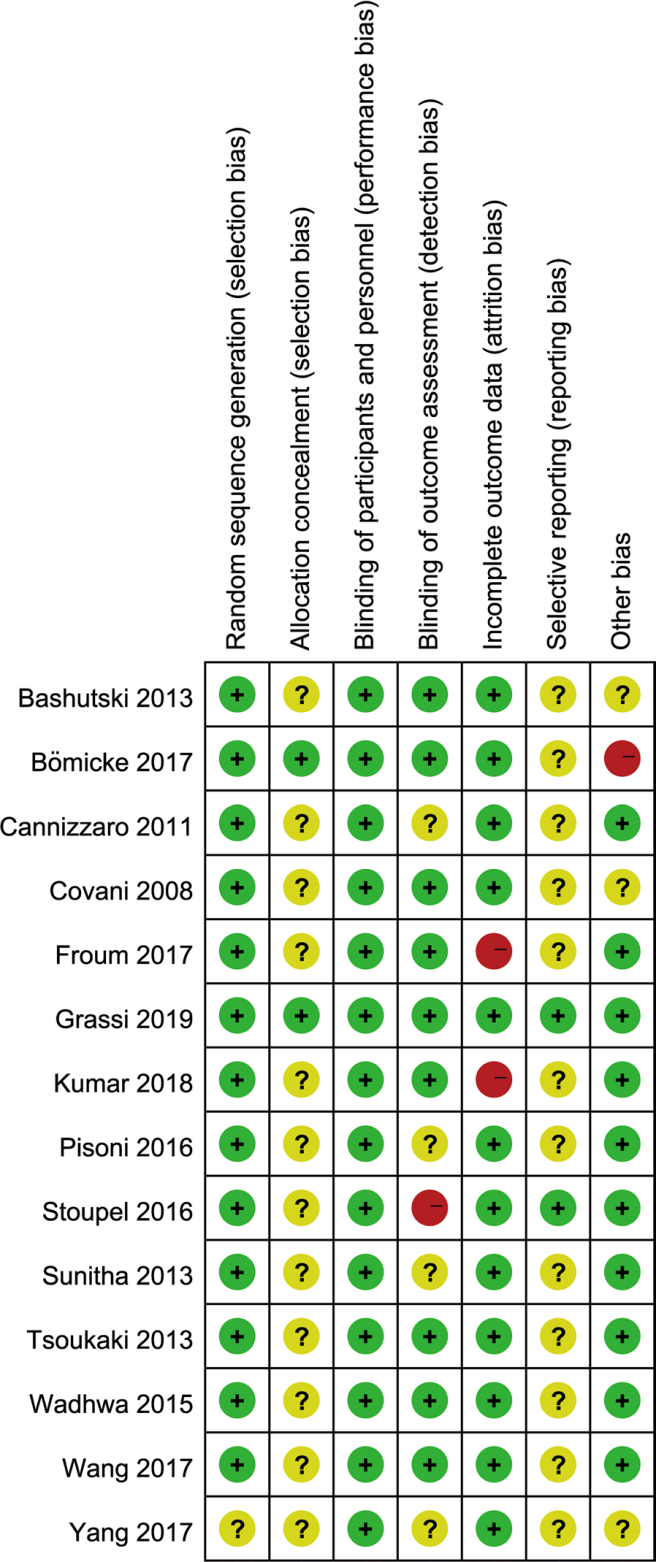
Risk of bias summary: each risk of bias item for each included study

The risk of bias in the included RCTs was relatively high (Fig. 2). One study was at unclear risks of random sequence bias and the rest of the studies were at low risks of bias. Two studies were at low risk of allocation concealment while the rest 12 studies did not mention the allocation methods. Since the blinding of participants and personnel was hardly in practice, all of the studies were recognized as low risks of blinding of participants and personnel. Two studies had more than 10% of the patients lost to follow-up, whereas the data in the other 12 studies were comparatively complete. As for the selective reporting bias, after thorough researching, two studies have registered their trials and reported the results according to the registration. One study was at high risk of bias as it compared the flapless surgery with one-piece implant placement and flap surgery with two-piece implant placement. However, no study has high risk of bias in more than one bias item (Fig. 3).

### General outcomes

**Fig. 4:**
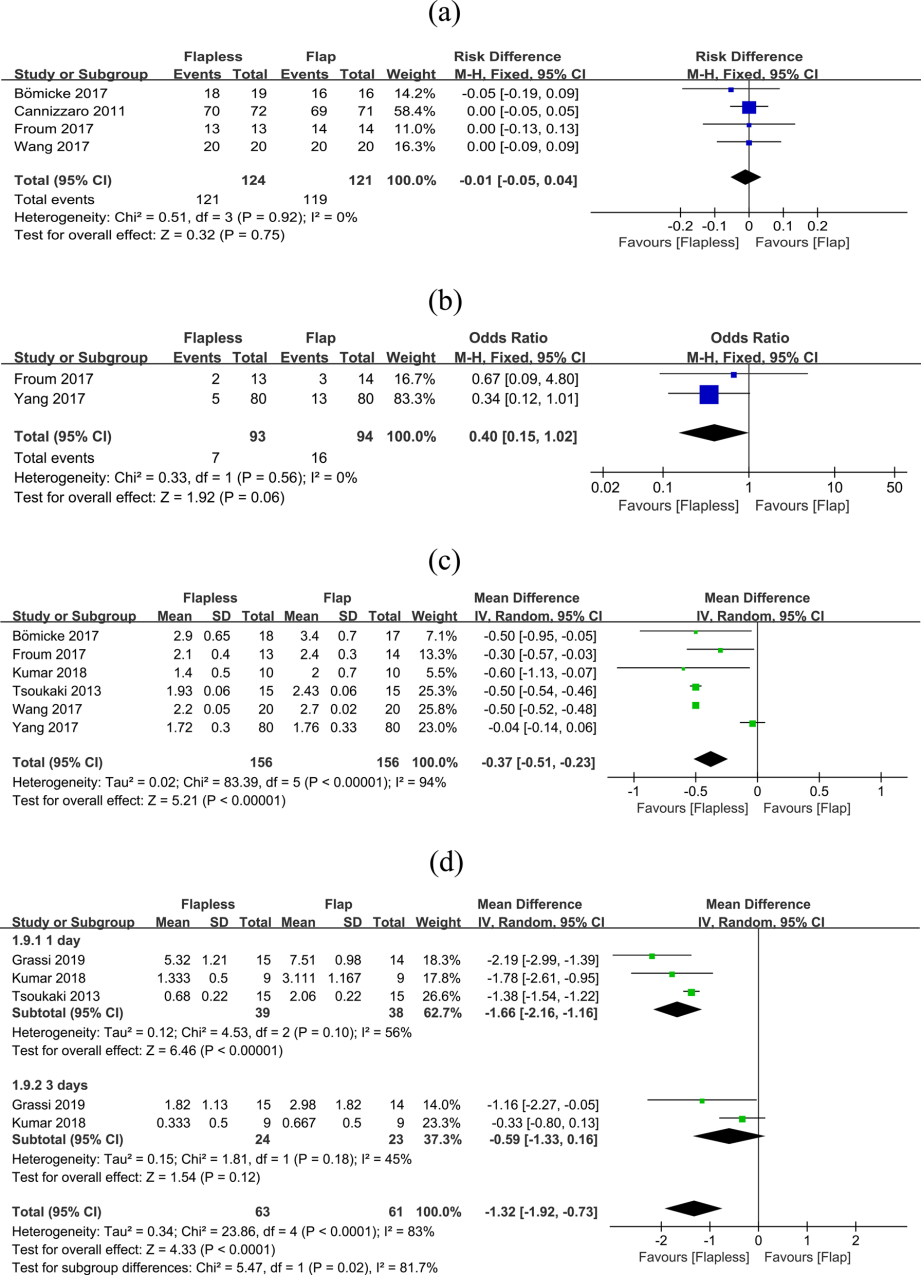
Comparisons of general outcomes after flapless and flap techniques. **a** SR, **b** BOP, **c** peri-implant PD, **d** VAS

The general outcomes compared the SR, BOP, PD and VAS of both techniques. No significant differences were detected between the flapless and flap approaches in SR (flapless VS flap: RR = -0.01, 95% CI (− 0.05, 0.04), *P* = 0.750) (Fig. 4a). Two studies were included to evaluate the BOP index. There was no significant difference between two techniques (flapless VS flap: OR = 0.4, 95% CI (0.15, 1.02), *P* = 0.060) (Fig. 4b). As for peri− implant PD, only results up to 3 months were included to compare the effects on soft tissue. Surprisingly, the flapless procedure resulted in a less peri-implant PD than the flap one and the difference were significant (flapless VS flap: WMD = − 0.37, 95% CI (− 0.51, − 0.23), *P* < 0.001) (Fig. 4c). VAS between the two groups was significantly different on both day 1 and day 3. On the first day, patients felt more pain and discomfort with worse experience after the flap procedures (flapless VS flap: WMD = − 1.66, 95% CI (− 2.16, − 1.16), *P* < 0.001). The difference was insignificant after 3 days (flapless VS flap: WMD = − 0.59, 95% CI (− 1.33, 0.16), *P* = 0.120). And the difference was still very significant overall (flapless VS flap: WMD = − 1.32, 95% CI (− 1.92, 0.73), *P* < 0.001) (Fig. 4d).

### Aesthetic outcomes

**Fig. 5:**
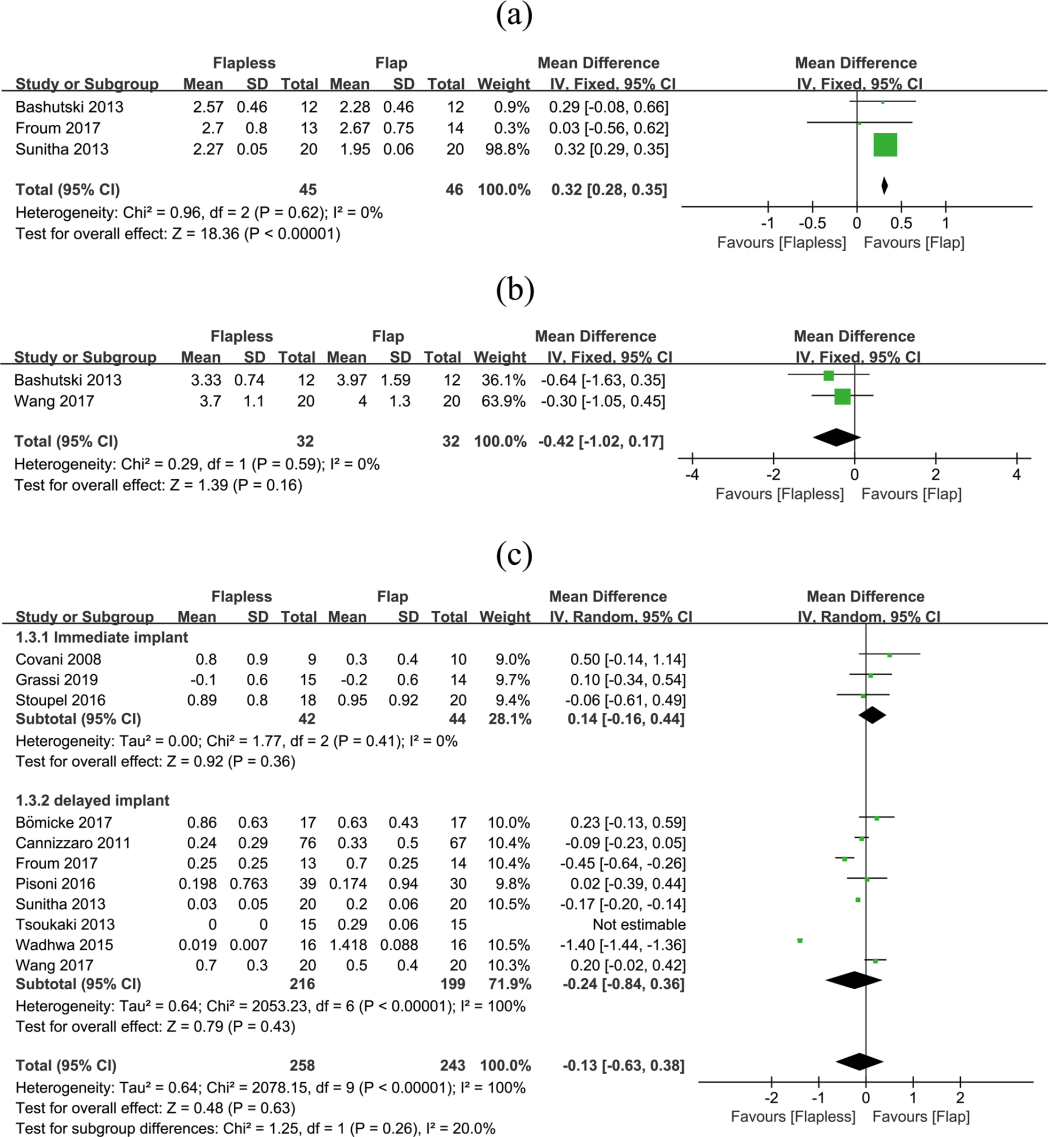
Comparisons of aesthetic outcomes after flapless and flap techniques. **a** PPI, **b** KM width, **c** CBL

Aesthetic outcomes put emphasis on the peri-implant soft and hard tissues. Only three studies have evaluated PPI. The flapless technique lead more gingival presentation compared to the flap technique (flapless VS flap: WMD = 0.32, 95% CI (0.28, 0.35), *P* < 0.001) (Fig. 5a).

KM width was further compared between 2 studies containing 64 implants. No significant difference was detected between the two procedures (flapless VS flap: WMD = − 0.42, 95% CI (− 1.02, 0.17), *P* = 0.160) (Fig. 5b).

The results of CBL were classified into two subgroups: immediate implantation and delayed implantation. Either the immediate subgroup (flapless VS flap: WMD = 0.14, 95% CI (− 0.16, 0.44), *P* = 0.360) or the delayed implant subgroup (flapless VS flap: WMD = − 0.24, 95% CI (− 0.84, 0.36), *P* = 0.430) would lead to insignificant differences in CBL. As can be seen in Fig. 5c, there was no significant difference in crestal bone changes between two groups overall (flapless VS flap: WMD = − 0.13, 95% CI (− 0.63, 0.38), *P* = 0.630).

### Risk of bias across studies

The publication bias was estimated via Egger’s test. No significant publication bias were detected among the studies in these analyses (flapless VS flap: SR: coefficient = − 0.074, 95% CI (− 0.487, 0.340), *P* = 0.523; peri-implant PD: coefficient = 2.202, 95% CI (− 4.245, 8.650), *P* = 0.397; VAS: coefficient = − 1.811, 95% CI (− 10.462, 6.841), *P* = 0.229; PPI: coefficient = − 0.620, 95% CI (− 6.070, 4.830), *P* = 0.385; CBL: coefficient = 3.435, 95% CI (− 11.831, 18.701), *P* = 0.618). The results of BOP and KM width were not evaluated by publication bias due to the limited number of included studies (*n* = 2).

### Additional analysis

**Fig. 6:**
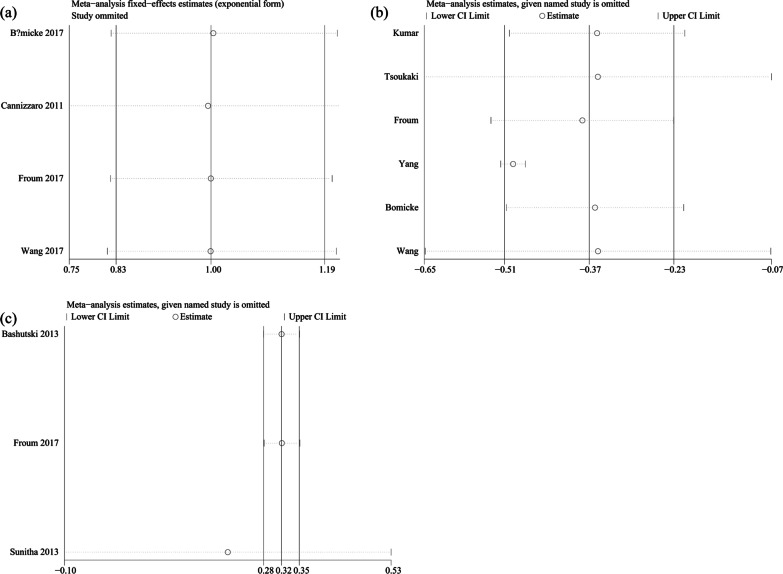
Sensitivity analysis of each evaluated outcome: **a** SR; **b** peri-implant PD; **c** PPI

Sensitivity analyses were then conducted. The results of SR and peri-implant PD meta-analysis were not affected when any included study was omitted (Fig. 6a, b). The 95% CI of the data in one study comparing PPI was not within the upper and lower limits, while the exclusion of this data did not affect the final results (Fig. 6c). This also indicated a great deal of certainty.

Subgroups analyses of VAS scores and CBL were also conducted and the results were clarified above.

## Discussion

This meta-analysis included 14 RCTs analyzing clinical performances of 720 implants over a follow-up of 3 months or more. Quality of the included studies was generally low. Based on the results of analyses, the flapless technique showed better effects on the gingival papillae preservation, induced less peri-implant probing depth, and brought less pain and discomfort, compared with the flap technique. However, flapless and flap techniques showed comparable effects over SR, BOP, KM width, and CBL.

Flapless and flap procedures are both widely applied in implanting [39]. As high survival rate is one basic requirement for dental implanting, we have compared SR of the two approaches and found no significant difference, which was consistent with previously published reviews [9–11]. In most of the included studies, patients were prescreened using radiographs to make sure that there was enough bone volume for implantation. In that case, SR would be guaranteed in both groups.

The incidence of peri-implantitis was then evaluated by BOP [40]. The overall BOP of the two groups were both low and the between-group difference were insignificant. The follow-up time of the two included studies were 1 year and 8.2 years, respectively, indicating that neither flapless nor flap technique may show side effect on peri-implant tissues in the long term.

After implant surgery and mucosa injury, there is about 6–8 weeks of wound healing and maturation [41]. This meta-analysis only included studies comparing the peri-implant depth over 3 months, which is long enough for the full recovering of gingival mucosa. The soft tissue seal around implants was essential as it makes a barrier for the invasive bacteria and other foreign microbes. However, an overlong soft tissue length around implants (peri-implant PD > 5 mm) indicates the high risks of peri-implantitis [42]. In all of the studies, the mean PD was within 4 mm, which indicated no significant risks of peri-implantitis. As can be seen in Fig. 3c, when the gingiva was fully recovered, PD in flapless group was deeper than the flap one. This was in accordance with an in vivo canine study, which reported a 0.7-mm deeper probing depth after flap implantation compared with the flapless technique [43]. The increased PD may be due to the severer inflammatory infiltration and fibroplasia around incisions in the flap group.

VAS is considered as a valid tool for assessing dental perception [44]. Within the first day after surgery, the VAS was the highest but still within the acceptable range of pain scales for the two approaches. Within 1–3 days after surgery, the mean VAS was below 1 in both the two groups. Patients would experience less pain and discomfort in the flapless approach in the first day. However, flapless and flap approaches resulted in comparable pain and discomfort 3 days later. Therefore, the flapless technique is a minimally invasive approach and could offer patients a better surgery experience.

The degree of gingival presentation is regarded as an important aspect of aesthetic effect after implantation and graded as a component in the “pink aesthetic scores” [45, 46]. PPI was measured only in three studies that compared the delayed implant surgeries and only two of them compared it in the aesthetic regions. The flapless approach has led more gingival papillae presentation compared with the flap one and the difference was quite significant. The vertical distance from the alveolar crest to contact area of two adjacent crowns is considered as the most significant factor for gingival papillae presentation [47]. When the distance was lower or equal to 5 mm, the papillae was presented in 98% of the ceases. With the vertical distance rising, PPI continuously reduced [48, 49]. In the delayed implant procedures, the flap technique would involve a horizontal incision between the adjacent teeth, the operation and the tension in suture may impair the recovering of gingival papillae. Besides, elevating mucosa flaps would cut the blood supply from the periosteal and mucosa and thus may cause the interdental alveolar crest absorption.

Keratinized mucosa is essential for the health and aesthetics of peri-implant tissue in anterior regions. When only 3–4 mm keratinized mucosa was presented on the buccal side of the gingival, a lateral flap advancement was indicated for further surgery. Sites with KM width < 2 mm showed a higher chances of suppuration and marginal bone loss [50]. Nonetheless, chances are really high that the patients have gummy smile issues when an excessive KM width is presented in the maxillary anterior region [51]. Flapless techniques for delayed implant placement require a circumferential excision of keratinized mucosa at the implant site. Even though a part of the keratinized mucosa was excised in the flapless group, no significant decrease in KM width was detected.

CBL was detected for diagnosing peri-implantitis and evaluating the aesthetic effects. Greater CBL might indicate the subsequent marginal recession in the long term and increasing aesthetic risks. Most of the selected studies measured the CBL by radiographic images. However, some included the immediate implant treatments and some included delayed implant treatments. As the implant timing would influence CBL and there was analysis indicating that immediate implant placement would result in more marginal bone loss, we conducted subgroup analysis [52]. However, in the two subgroups, the difference of CBL was not significant. A previous study has demonstrated that the partial-thickness flaps would lead to regenerated bone in 3–7 days, while full-thickness flaps lead to no bone regeneration [53]. Although most of the included studies have not stated whether the partial-thickness or full-thickness flaps was applied in their studies, no difference of marginal bone height was detected.

This was the first systematic review and meta-analysis of only RCTs to assess both the general and aesthetic outcomes between flapless and flap implant techniques. We included only RCTs to increase the robustness of the results. Aesthetic effects were compared for the first time in the review. However, there were some shortcomings in the analysis. Flapless techniques, including punch, drill preparation and IIP, were all included in our study to get a comprehensive summary of the effects of flapless technique. The geometry and biological situations of the soft and hard tissues between the IIP and punch procedures might be different. Nonetheless, we conducted subgroup analyses between immediate and delayed (punch/drilling) flapless procedures and flapped procedures in our study when the number of included studies in each subgroup was sufficient (n ≥ 2). Also, more sensitivity analyses have been applied and the exclusion of the studies that used immediate or delayed implant placement did not affect the final results. Few studies compared the aesthetic effects of the two groups. Even when the aesthetic outcomes were taken into consideration in some trials, there are no sufficient data to thoroughly elucidate the outcomes. More thorough investigations are needed to compare the aesthetic effects, such as soft tissue contour around implants, soft tissue level and alveolar process deficiency.

## Conclusions

This meta-analysis revealed that flapless techniques could help to preserve gingival papillae and reduce the pain and discomfort after surgeries. Besides, the flapless procedure showed less peri-implant probing depth. Flapless technique would be recommended in implanting when there is enough soft and hard tissue dimension. More high-quality and aesthetics-related RCTs are needed to draw a more comprehensive conclusion.

## Data Availability

Not applicable.

## Abbreviations

RCTs: Randomized controlled trials
SR: Survival rate
BOP: Bleeding on probing
PD: Probing depth
VAS: Visual analogue scale
PPI: Papillae presentation index
KM: Keratinized mucosa
CBL: Crestal bone loss
RR: Risk ratio
OR: Odd ratio
CI: Confidence interval
WMD: Weighted mean differences
PRISMA: Systematic Reviews and Meta-analyses
PICOS: Participants–Interventions–Comparisons–Outcomes–Study Design
IIP: Immediate implant placement
NR: Not reported
